# Strategies for patient empowerment through the promotion of medicines in Israel: regulatory framework for the pharmaceutical industry

**DOI:** 10.1186/s13584-017-0175-y

**Published:** 2017-09-29

**Authors:** Eyal Schwartzberg, Zohar Barnett-Itzhaki, Itamar Grotto, Eli Marom

**Affiliations:** 1 Pharmaceutical & Enforcement Divisions, Ministry of Health, 39 Yirmiyahu St, Jerusalem, Israel; 2 Ben-Gurion University of the Negev, Beer sheva, Israel; 3 Arnold and Marie Schwartz College of Pharmacy, Long Island University, Brooklyn, USA; 4 Public Health Services, Ministry of Health, Jerusalem, Israel; 5 Bioinformatics department, school of life and health science, Jerusalem College of Technology, Jerusalem, Israel

**Keywords:** Adherence, Medications, DTCA, DAC

## Abstract

The correct and rational use of medications can have a positive direct impact on disease outcomes, as well on the utilization of the health system resources. Unfortunately, 50% of the patients do not take their medications as prescribed, largely due to lack of patients’ understanding of their medical condition, as well as the lack of reliable medicine information.

There are multiple strategies implemented in many countries to tackle this challenge including: disease awareness campaigns (DAC) to raise the public awareness to specific diseases, direct-to-consumer advertisement (DTCA) to raise the public awareness to prescription medicines, specific treatments and over-the-counter (OTC) products to improve the accessibility of patients to specific medicines.

Prior to 2013, the Israeli policy prohibited prescribing medication advertising and prevented the flow of information from pharmaceutical companies to the patient. In the last five years, the Pharmaceutical division in the Israeli Ministry of Health, as part of the “empowering the patient” agenda, has taken new innovative approaches to raise public awareness to diseases, medications and appropriate usage, as well as promotion of information to improve patient adherence to the prescribed medication.

This paper elaborates on the aforementioned strategies implemented in developed countries, and specifically focuses on newly implemented strategies and regulations in Israel regarding pre- and post-prescription information, to improve patient appropriate utilization and adherence to medication.

## Background

Medicines have the potential to benefit the individuals who use them. Nevertheless, the use of medicines may be associated with undesired adverse effects. The decision to prescribe and take prescription medicines, as well as OTC medications, and subsequently achieve desirable therapeutics outcomes, requires additional knowledge and expertise, not only by health care providers but also by the patients.

Available information about health conditions, medicines, and their use can principally be obtained from two main sources: (a) healthcare providers: primarily physicians, pharmacists and nurses, and (b) the pharmaceutical industry. Additional sources of information are available in the media including: press, internet, social media, medical and general literature, etc. The first two sources of information are regulated in Israel by the Ministry of Health (MoH).

Prior to 2013, the MoH focused mainly on prohibiting prescribing medication advertising, while allowing OTC advertising only. General information about disease awareness and the contact between pharmaceutical companies and patients were not clear. Due to the rise of social networks and the penetration of the internet, the MoH had decided to provide guidance to involved stakeholders and especially the pharmaceutical companies in order to avoid inaccurate and false information.

The law recognizes that medicines should not be handled as an ordinary consumer product. This is achieved by a variety of restrictions, in particular prohibiting advertising to general public. Such limitation of pharmaceutical advertising is due to the concern that misleading or inaccurate advertising may lead to misuse of pharmaceutical products, including increase pressure on health care providers to prescribe specific drugs, which may put patient’s health in risk.

According to The Israeli ‘Regulation of the Pharmacists’ (article 28 to the pharmacist regulation, medicinal products), advertising of non-prescription pharmaceuticals is allowed only after authorization is granted by the Pharmaceutical Division (PD) of the MoH, while direct-to consumer advertising (DTCA) of prescription pharmaceutical products is illegal, unless approved by the director general of the MoH.

One of the major goals of the Israeli MoH, as set by the “pillars of fire” (the ministry credos), is centralizing patients’ needs and care, empowering their knowledge, as well as fulfilling their rights for high quality standard of care. Based on this pillar, the efficient use of pharmaceutical products was set as a key objective by the PD of the MoH. The PD is the governing authority for the pharmacy profession in Israel and serves as the regulating body of the pharmaceutical industry. One of the major methods to enhance appropriate use of medicines is to improve patient adherence. Adherence could be defined as the process by which patients take their medications as prescribed [1]. This process could be divided into three stages: **initiation** i.e.: filling the prescription, collecting the prescribed medication and starting treatment, **implementation:** how the patient is taking his medications, and **discontinuation** of treatment [2]. The challenge of non-adherence is a global issue that has both direct negative outcomes on patients’ health, as well as additional increase on expenditures and resources by the health system. According to the world health organization (WHO), approximately 50% of the population does not use their medication as prescribed by their physician [1]. Non-adherence may stem from number of causes, such as lack of understanding for the treatment importance, barriers to accessibility to healthcare, side effects, financial reasons, early discontinuation and other issues. Non-adherence is a multifaceted challenge that requires significant effort to overcome. Currently there is no available information regarding the rates of non-adherence and their causes in Israel.

Adherence issues are also seen in over the counter (OTC) medicines, where patients treat themselves for minor ailments and conditions. This in turn may lead to additional drug related problems such as possible overdose.

The following paper describes international and Israeli approaches to improving adherence by raising public awareness to diseases, medications and their usage. The paper focuses on the impact of Disease Awareness Campaigns/Communications (DAC), promotion of information to improve adherence in patients who were prescribed a medicine (Adherence Campaigns - AC), OTC Direct to consumer advertisements (OTC - DTCA) and the role of the pharmacist in this process.

### Regulation of DAC and DTC and DTCA in developed countries

Currently, direct-to consumer advertising (DTCA) and promotion of prescription drugs is permitted only in the USA and New-Zealand. The following section focuses on different approaches and policies in developed countries regarding DAC, DTC and DTCA.

### USA

As from 1985, The Federal Food, Drug, and Cosmetic Act permits advertising of prescription medicines, as long as the advertisements are accurate and not misleading. In 1997, U.S. Food and Drugs Administration (FDA) published a rule obliging companies to offer a detailed list of side-effects in their infomercials (long format television commercials). The main FDA guidance on DTCA was issued in 1999, *Guidance for Industry, Consumer-Directed Broadcast Advertisements* [3, 4]. The American Medical Association (AMA), additional professional bodies, and politicians, have called for a ban on DTCA of prescription medicines and medical devices. However, prohibiting DTCA would require an act of Congress to change the Federal Food, Drug, and Cosmetic Act and would also raise complex freedom of speech issues [5].

According to the FDA, DAC can provide important health information to consumers and health care practitioners, and can encourage consumers to seek, and health care practitioners to provide, appropriate treatment. The guidance prohibits mentioning a particular drug or device [6].

### New Zealand (NZ)

The New Zealand authority never enacted legislation about DTCA, seemingly more by accident than design, because prescription medications were simply not being advertised in 1981. A consultation document from 2006 reviewed the policy debate on DTCA in NZ, outlining the current policy for DTCA and the Therapeutic Products Advertising Code [7]. DTCA was unanimously supported by advertising agencies and pharmaceutical companies and no further actions were taken by the government, let alone any legislative change to prohibit DTCA [8].

### European Union

The EU legislation prohibits the advertising of prescription-only medicines directly to patients and consumers. Directive 2001/83/EC of the European parliament relates to medical products for human use and states that EU members must prohibit the advertising to the general public of medicinal products which are available by prescription only [9]. Council Directive 89/552/EEC on the pursuit of television broadcasting activities, prohibits the television advertising of medicinal products which are available only by prescription in the member state within whose jurisdiction the television broadcaster is located [10].

### UK

The British Authority has the most comprehensive policy on DAC, which are outlined in *the Blue Guide:* advertising and promotion of medicines in the UK [11]. Medicines advertising in the UK are regulated by a combination of European and national legislation. These regulations clearly state that any advertisement to the general public which is likely to lead to the use of a prescription only medicine is prohibited.

The *Blue Guide* encourages DAC, but does not allow the use of brand names or restrict the range of treatments described in the campaign. The provided information should help the public to be able to recognize the disease or its risk factors, and take preventive measures, if appropriate. Additionally, DAC should point out what the patient needs to do immediately, if necessary, and where to get appropriate advice on management options [11].

### Australia

The *Regulations of therapeutic goods advertising in Australia,* prohibits DTCA of prescription only medicines. Advertising of prescription only medicines to health professionals is permitted and is regulated by a self-regulatory scheme operated by *Medicines Australia*, an Australian pharmaceutical manufacturers association. In addition, information about a medical condition or specific treatment (not brand name) may be distributed to the general public, as a “community service” [12].

### Canada

The Canadian *Food and Drugs Act and* the *Food and Drug Regulations* prohibits DTCA for health products (including medical devices) which make claims to treat, prevent or cure any of the serious diseases. In addition, these regulations prohibit DTCA beyond the drug’s name, price and quantity. DTCA of Prescription medicines and DAC in Canada are regulated by the two agencies: *Advertising Standards Canada* and *Pharmaceutical Advertising Advisory Board* [13, 14].

### Examples for the impact of DAC and DTC around the world

Promoting diseases to sell medicines is a common and venerable practice among pharmaceutical companies that try to expand their market size by implying that large parts of the population suffer from the disease or condition. Although some people may legitimately suffer from a particular disorder and require medicine treatment, others might be mistakenly diagnosed with a disorder they do not actually have, or start taking medications that might not ultimately benefit them [15]. DAC have the potential to influence consumer behavior to prefer a specific medicine, but also to influence physicians to prescribe a specific medicine to his or her patients.

In 1995 researchers analyzed the response to DTCA of a new migraine medicine (Imitrex, active ingredient sumatriptan succinate) and showed an association between the advertising campaign and a significant increase in new prescriptions for that medicine. The study showed that men and high incomes individuals were significantly more likely to respond to DTCA [16].

A Dutch campaign for terbinafine that was initiated in 2000 by Novartis, included television advertisements advising people with onychomycosis to consult their general practitioner. The *Dutch Society of General Practitioners* objected to this campaign but recommended terbinafine, even though another medicine (itraconazole) was also available as an oral treatment for onychomycosis. Novartis discontinued the campaign two years later. The unbranded campaign resulted in a considerable increase in terbinafine prescription rate, and in a slight decrease in itraconazole prescription rate, despite the fact that the campaign did not specifically mention terbinafine. Furthermore, the campaign was successful in motivating people to seek care for onychomycosis which is strongly suggested by the concurrent increase in the consultation rate for onychomycosis. After the campaign was discontinued in 2002, rates of consultations and prescriptions dropped [17].

There are evidence that disease awareness advertisements (DAA) increases awareness of the advertised health conditions. A recent content analysis examined the prevalence of DAA in top circulating Australian women’s magazines and concluded it constituted approximately 12% of all therapeutic advertisements (DAA, DAC and others). This finding suggests its potential exposure is at least moderate [18].

In fact, DAC are controversial: several scientists and physicians consider them as medicine mongering and claim that they turns healthy people into patients, waste precious resources, and causes iatrogenic harm [19]. They also claim that the pharmaceutical industry invents new markets only to make more money [20]. For example, in 2003 the pharmaceutical company *GlaxoSmithKline* launched a massive campaign to promote awareness of “restless legs syndrome”. The campaign included press releases which suggested that a known Parkinson medication (Requip (Ropinirole)) is also suitable to treat restless leg syndrome. The campaign included news articles and radio shows that exaggerated the prevalence of the disease and the need for treatment. All of the above contributed to over-diagnosis of the syndrome and over-usage of this medication, while according to physicians the recommended treatments for the syndrome include stretching exercises and less caffeine for intermittent disease, and various prescription medications (e.g. benzodiazepines and dopamine agonists) for daily symptoms [21].

An example of a potentially problematic aspect of DTCA is an advocacy group for people with ADHD (named CHADD – Children and Adults with ADHD) that is partially supported by the pharmaceutical industry. CHADD acted in the past as the lead editorial consultant of a special issue on ADHD in *Health in Action*, a quarterly publication of the *American School Health Association*, and currently undertakes educational programs for teachers. This may influence teachers who have a formal role in the diagnosis of ADHD in the U.S, U.K and Australia, and may increase a false diagnosis of ADHD and over-usage and unnecessary usage of ADHD medications [22].

However, there are significant advantages to DAC, which can improve the public health and even save lives. One such example is a DAC implemented in rural areas of East Africa (Kenya, Uganda and Tanzania) to promote the use of artemisinin-based combination therapy (ACT) to treat Malaria. The programs assessed retail sector ACT subsidies combined with supportive interventions that included community awareness and mass media campaigns. The programs increased the usage of ACT in children aged 0–5 years, and are believed to increase the usage by 19% - 41% over a one year period. These programs also reduced the use of older antimalarial drugs among febrile children aged 0–5 [23].

Another example is the Mycobacterium ulcerans infection that can cause Buruli ulcer, one of the most rapidly emerging diseases in West Africa in recent decades. Recent research showed that antibiotic therapy with rifampin and streptomycin may reduce the extent or prevent excision when initiated during the early phases of the disease. Analysis of epidemiological data from existing Buruli ulcer control programs in West Africa indicate that active public awareness campaigns are successful in increasing awareness and understanding while decreasing delays in treatments and disease progression [24].

### Regulation in Israel

The Israeli national health insurance (NHI) system provides universal coverage. Every citizen or permanent resident of Israel can choose from among four competing, non-profit health funds, called Health Maintenance Organizations (HMOs). The HMOs must provide their members with access to a statutory benefits package. [25]. More than 4000 medications are registered for use in Israel. The Israeli MoH website provides comprehensive data on all the registered medicines in Israel.

Various efforts are under way to promote the use of generic medications and of the lowest cost pharmaceuticals. By law, generics may be prescribed and dispensed generically and substituted by the HMO for brand-names medication whenever clinically appropriate.

According to the Israeli Pharmacists Regulations (article 28), advertising of non-prescription pharmaceuticals is allowed only after a permit by the PD, practically making the DTCA of prescription pharmaceuticals illegal. The prohibition to advertise applies to the registration holder as well as to any individual or third party.

DTCA in Israel is allowed for OTC medications, in order to empower the patients and enable them purchasing medications, in an informed manner, without the need to visit or consult their physician. Of note, most of the OTC medications are taken to treat minor medical problems.

### Currently available medical information for patients in Israel:

In the past 5 years, the PD initiated several services and enterprises in order to improve patients’ access to pharmacological information. Of note, similar initiations and services are enforced worldwide, especially in the countries which are recognized by the Israeli legislation: EU countries, Switzerland, USA, Canada, Australia, New Zealand, and Japan.

### Package & multilingual patient leaflet

The Hebrew language is spoken by the majority of the Israeli population. According to a public survey from 2011, 18% of the Israelis speak Arabic, 15% speak Russian, 2% speak English and 8% speak other languages (including Amharic) [26]. In accordance with the pharmacist ordinance, and the pharmaceutical regulations and procedures, the outer package of every pharmaceutical product in Israel must be labeled in four languages: Hebrew, Arabic, English and Russian. The patient’s leaflet must be written in three languages for prescription medicine and OTC (Hebrew, Arabic and English), and four languages for General Sales List (including Russian). The physician leaflet must be written in English. Such information is determined and validated in the registration process and has a pre-defined format [27].

### **The Israeli drug registry** [28]

The Israeli Drug Registry website consists of all commercial pharmaceutical products and the terms of registration, including the commercial name, the manufacturer, the active ingredients, dose, indications, the physician leaflet, the patient’s leaflet, a picture of the packaging, and an updated medication price. It includes additional information items such as information regarding gluten free medications. The website is updated every month and is widely used by patients, health care professionals and the general public.

Recently, a new query regarding the inclusion of commercial pharmaceutical products in the national list of health services (“the health basket”) has been added to the Israeli drug registry database. Future development will address the issue of generic substitution, information regarding optional actions (such as medication grinding), as well as images of all registered drugs.

### OTC advertisements

As already been mentioned, the Israeli law prohibits DTCA of prescription medications but allows the advertisement of non-prescription pharmaceuticals (with approval of the PD). Prior to 2013 there was only general guidance regarding advertising policy of medicines in Israel, in which only OTC medications were allowed to be advertised. Thus, without official guidance, the pharmaceutical companies used various marketing strategies which were controversial and unregulated. In 2013, new regulations for OTC advertisements were issued, and consequently the number of applications for approving OTC advertisements increased by more than 40% in the last three years (see Table 1).

**Table 1:** Number of applications for approving OTC advertisements (Source: PD)

Year	Total number of applications
2014	420
2015	533
2016	600

### New regulation and available medical information for patients in Israel: DACs and non-promotional medical information for patients to promote adherence

After internal discussions within the PD and a thorough review of similar international policies and activities, it was decided by the PD to set comprehensive guidelines for the pharmaceutical companies in Israel for the purpose of disseminating disease and medicine information to the general public. These guidelines took into consideration the fact that patients are exposed to medical information from various sources, some of which are not accurate and even biased or dangerous. As such, the guidelines aimed for balanced, non-promotional information and knowledge about disease awareness and information regarding medication used by patients. These guidelines and procedures were issued to the pharmaceutical industry, thus allowing them to engage in marketing activities in compliance with local regulations.

The MoH has established two new information channels: DAC (also known as procedure 134 DAC) which was published in 2014, and non-promotional medicine information for patients who are prescribed prescription only medications (also known as procedure 137: Promotion of Adherence) which was published in 2015.

### **Procedure 134 – Disease awareness campaigns (DACs)** [29]

The MoH and the PD provide national leadership in a broad range of public health domains, including empowerment of patients and rational use of drugs, health promotion, preventive health care and consulting on other medicine related issues.

The MoH has recently established regulations regarding DAC so that consumers can be empowered with information about the availability of treatments for diseases (medications or preventive treatments) in a manner that does not involve the promotion of a particular commercial product. For example: annual campaigns run by the MoH regarding influenza and recommendations for vaccinations, and campaigns and information regarding cancer, provided and run by the Israeli cancer association [30].

### **Procedure 137** [31]

This procedure regulates how pharmaceutical companies are allowed to encourage compliance among patients who have been prescribed medications (i.e. after the prescription is issued). In order to improve adherence with medicinal product, the patient receiving the prescribed drug can receive non-commercial information in various ways as specified in this procedure including digitally. The media and patient associations play an important role in increasing awareness to adherence with medicinal treatment. The information provided to the public must be balanced, factual, free of advertising and free of promotion to particular brand names. Of note, the information must not be intimidating and mustn’t create stress to the patients.

The objective of this procedure is to promote adherence to the prescription instructions, to improve patient’s collaboration with physician, to ensure the patient’s ability to manage the treatment prescribed to him, to supply the patient with scientific reliable information, and to increase awareness to reporting side effects (should they occur), in order to maximize the treatment’s efficiency and to protect the patient’s health.

The marketing registration (MAH) holder is responsible to comply with this procedure and must ensure conformity with all relevant laws. The information and instruction services specified in this procedure should be provided free of charge and under no condition or limitation. Patient consent must be attained per local Israeli law with emphasis on privacy issues and when applicable complying with database establishment and maintenance regulations.

After the patient gives his/her consent, a third party medical-information/adherence center, which is approved by the MoH and funded by the pharmaceutical company, contacts the patient. The center supplies two major services to the patients: (i) a call center that supplies information regarding the disease, the medication, and the correct way to take the medication. The telephone receptionist helps the patient with monitoring side effects. (ii) home visits for personalized training on appropriate usage of treatment, as well as reporting any adverse drug reaction that may be related to the product. Such services may be also deployed by the MAH, but a written justification for not employing third party should be sent to the ministry for consent.

It is important to keep in mind that some of the patients are people with disabilities who according to the law must be given access to the information **and as such, adherence support may be crucial to the successes of the treatment prescribed for them.**

### Analysis of the applications following procedures 134 and 137

We analyzed the applications submitted to the PD in 2015 and 2016 according to procedures 134 and 137. We used the *Anatomical Therapeutic Chemical* (ATC) classification index (WHO Collaborating Center for Drug Statistics [32]) in order to classify the pharmaceuticals.

**Fig. 1:**
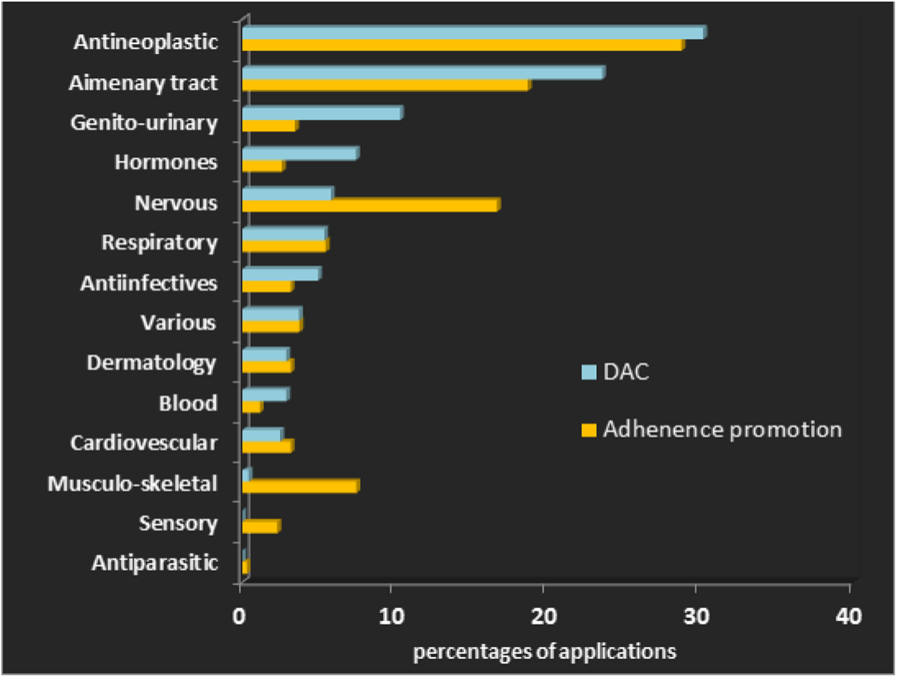
Promotion of adherence and DAC applications to MoH, percentages according to ATC pharmalogical groups. 348 DAC applications and 242 Adherence promotion applications were submitted the PD in 2015 and 2016. The figure shows these submissions according to their pharmacological groups

Three hundred forty-eight submissions according to procedure 134 were handled and 242 submissions according to procedure 137. The submissions were in a variety of pharmacological groups. Figure 1 shows the distribution of submissions per group.

Most of the activities were related to diabetes and cancer. Analysis of the results shows that the pharmaceutical companies’ activities were similar in both procedures (134 and 137), there is a correlation between the pharmacological groups of 134- and the 137-submission (Pearson correlation, R^2^ = 0.86).

### Procedure 112

This procedure (issued in 2013) defines the activities that need to be taken by pharmacists, while consulting and questioning patients during dispensing of medication in community pharmacies. The procedure is based on two legislations: (a) The patient rights law (from 1996), according to which, patients should receive adequate medical treatment, while pharmacists are defined as health care providers, and (b) The “Pharmacist ordinance” which defines the information that should be given to patients with the medicinal product. The procedure emphasizes which information should be provided to the patients by the pharmacist while dispensing OTC and prescription only medicines [33].

### “Trufa to go” application

Recently the PD launched a software application aimed at promoting rational and safe use of OTCs and General Sales List products. This application enables the patient to search the database by product trade name or symptoms experienced, and to scan the package barcode for product information. Information available for patients include: product picture, product information leaflet, indication, package size, indication and price. The application is being updated continually and is available for download free of charge from Google Play Market and Apple App Store [34].

## Discussion and conclusions

An effective pharmaceutical policy should ensure that patients have access to reliable, scientific, evidence based and relevant information. One of the major challenges of the Israeli MoH is to focus on the patients’ needs, to empower patients and to provide them with the necessary pharmaceutical knowledge. As such, an effective pharmaceutical policy should also concentrate on chronic patients treated with multiple drugs. These patients require more attention in terms of detection of side effects. Thus, the MoH preemptively published procedures promoting the provision of information to patients, using a variety of media and approaches: websites and a cellular application, in addition to new innovative regulations. These issues are also emphasized in a circular published by the Israeli MoH medical administration in 2013 [35].

The pharmacist’s role in dispensing medications and providing information is essential for safe, effective, and successful treatment. To implement this, the MoH uses various types of legislations regarding both dispensing medications and providing information for chronically ill patients. Additionally, the PD published the aforementioned procedure (134, 137) for patients requiring further guidance, support and training.

An innovative approach promoted by the MoH includes the pharmaceutical industry in policy efforts aimed to improve adherence at the post prescribing phase. The pharmaceutical industry has a clear financial incentive to increase its sales rate. Following the guidelines issued by the MoH, will benefit the industry by allowing them to keep investing in marketing, but at the same time to empower the patients. Providing patients with reliable and useful information will allow them to better adhere to recommended treatment regimes, achieve desired clinical outcomes, and most importantly - improve their health. An additional added value to implementing this new patient-oriented policy is in lowering the risk for unnecessary medico-legal litigation by improving the accuracy of the information reaching the patients. The Israeli experience suggests that the pharmaceutical industry has quickly and efficiently adopted the regulations and implemented them successfully.

In addition, it is important to safeguard the interests of the patients and to ensure the system’s transparency, reliability, and integrity. Hence, DTCA of prescription medications is not legal in Israel and physicians in Israel must not be paid for prescribing specific medications by law. Furthermore we believe that DTCA may lead to unwanted “disease mongering”, increased pressure on healthcare providers to prescribe unnecessary treatments as well as providing the patients with non-accurate and missing information regarding their condition. These may lead to unnecessary increased pressure on the health system as well as on its expenditures.

Non-adherence is a significant issue that has direct negative outcomes on patients’ health. Currently there are knowledge gaps regarding the rates of non-adherence in Israel. In order to fill the gaps, more research is needed: collaboration with the HMOs that collect data regarding subscriptions of medications and how many of them were actually purchased, in addition to national surveys to reveal the rates of non-adherence due to implementation problems and discontinuation of treatment.

It should be noted that the physicians are responsible for monitoring the patient’s adherence and that procedure 137 (and the industry’s involvement) is intended to support this process rather than replace it. in the last 5 years there has been a significant progress in regulation of medicine advertising and in patient’s information. Furthermore, there is an ongoing process of learning and improving: the MoH keeps on monitoring and self auditing its’ procedures, compares the regulation to other developed countries, continues holding discussions with involved stakeholders, and keeps on developing and amending its policy in this matter.

In our opinion it is highly important for the regulator to issue guidelines and procedures to regulate the transfer of accurate, reliable and useful information to patients. Such means should address key stakeholders in the process of medication adherence: healthcare providers, patients and the pharmaceutical industry.

## Abbreviations

ACT: Artemisinin-based combination therapy
AD: Adherence Campaigns
AMA: American Medical Association
ATC: Anatomical Therapeutic Chemical
CHADD: Children and Adults with ADHD
DAA: Disease awareness advertisements
DAC: Disease awareness campaigns
DTCA: Direct-to-consumer advertisement
FDA: Food and Drugs Administration (USA)
HMO: Health Maintenance Organization
MAH: Marketing registration
MoH: Ministry of Health
NZ: New Zealand
OPDP: Office of Prescription Drug Promotion (USA)
OTC: Over the counter
PD: Pharmaceutical Division
UK: United Kingdom
WHO: World Health Organization
